# Inpatient Satisfaction with Nursing Care and Its Impact Factors in Chinese Tertiary Hospitals: A Cross-Sectional Study

**DOI:** 10.3390/ijerph192416523

**Published:** 2022-12-09

**Authors:** Mudan Yan, Mengjia Zhi, Yue Xu, Linlin Hu, Yuanli Liu

**Affiliations:** School of Health Policy and Management, Chinese Academy of Medical Sciences & Peking Union Medical College, 9 Dongdan Santiao, Dongcheng District, Beijing 100730, China

**Keywords:** quality of nursing care, patient satisfaction, Chinese, tertiary hospitals

## Abstract

Aims: To evaluate the level of patient satisfaction with nursing care in China’s major tertiary hospitals, and to explore patient and hospital level factors associated with patient satisfaction, in order to provide policy implications for the evaluation and improvement of nursing care, toward a patient-centered healthcare system. Background: Patient satisfaction with nursing care has been designated as a key measure of the quality of nursing care and is one of the main focuses of the current healthcare improvement campaign in China. Methods: We participated in the 2021 China National Patient Survey and designed and administered a survey instrument with five domains, to evaluate patient satisfaction with nursing care among 24,971 inpatients from 143 tertiary hospitals across 31 provinces in China. Descriptive analysis and binary logistic regressions were utilized to assess the level of satisfaction and identify key factors related to nursing satisfaction. Results: The overall satisfaction level is high, but satisfaction with health guidance is relatively low. Patients’ sociodemographic characteristics, including age, income, and education, are significantly associated with their satisfaction. Hospital characteristics, such as geographic location, type, and nurse-to-doctor ratio, significantly impact inpatient satisfaction with nursing care. Patients of hospitals in the eastern region, general hospitals, and hospitals with higher nurse-to-doctor ratios are more satisfied with nursing care. Conclusion: More attention should be paid to equitably allocating healthcare resources among different regions, improving the management and competence of non-general hospitals, and strengthening the recruitment and retention of the nursing workforce. Moreover, patient characteristics should be considered when evaluating patient satisfaction across hospitals. Patient and Public Contribution: These findings will help us better understand patients’ satisfaction regarding nursing care in Chinese tertiary hospitals and develop targeted interventions, to establish a patient-centered healthcare system.

## 1. Introduction

### 1.1. The Importance of Patient Satisfaction with Nursing Care

Nursing care is an integral component of medical care and is extremely important in a patient-centered healthcare system [1,2,3]. Nursing staff are professional caregivers available to patients around the clock; the primary sources of information to physicians regarding the conditions of patients; skilled practitioners in their own right, with the ability to perform the majority of direct care procedures; and often the first to identify and stabilize downturns in a patient’s condition [4,5,6].

Patient satisfaction with nursing care reflects the actual fulfillment of patients’ needs and expectations, which is a concrete criterion for evaluation of quality of nursing care [7,8,9]. It is widely considered to be a key element in the overall patient satisfaction and is important for health outcomes and hospital quality management [3,10]. Patients who are more satisfied with their nursing care are more likely to have a good relationship with nurses, follow medically prescribed regimens, and thus have more positive health outcomes [11,12,13,14]. Furthermore, measuring patient satisfaction with nursing care could help evaluate patient satisfaction; learn patients’ expectations, suggestions, and feedback; and provide crucial information for quality improvement [9,15].

### 1.2. Literature Review

In recent years, a large body of studies have been carried out to assess patient satisfaction with nursing care and its impact factors [16,17,18]. A number of survey instruments have been developed to measure patient satisfaction with nursing care from different perspectives. For instance, the patient satisfaction scale (PSS), built by Risser, is extensively used in the assessment of nursing quality. This scale is divided into three subscales: technical-professional area, interpersonal educational relationship, and interpersonal trusting relationships [19,20,21]. The La Monica-Oberst Patient Satisfaction Scale (LOPSS) is also employed to assess the quality of nursing care. It classifies customer preferences into three categories: dissatisfaction, interpersonal support, and good impression [22]. In a review of studies on the measurement of patient satisfaction with nursing care, researchers identified various factors that can be assessed by the patient, including the quality and quantity of nursing care, attitude, communication, physical environment and facilities, and outcomes [23]. Beyond that, scales suited to diverse national circumstances have been developed, such as the Newcastle Satisfaction with Nursing Scales (NSNS) and its adaptions in different countries [24,25,26]. As for factors affecting inpatient satisfaction with nursing care, previous studies have explored the impacts of patient characteristics and hospital/care characteristics [7,24,27]. Regarding patient characteristics, sex, age, income, and duration of hospitalization are important impact factors affecting satisfaction with nursing care [28,29,30,31,32]. Regarding hospital/care characteristics, the hospital environment, quantity of nurse personnel, ward type, and behaviors of nurses are important impact factors affecting satisfaction with nursing care [25,33,34,35].

### 1.3. Research Gap in China

As part of the country’s ongoing health care reform, China is working to improve its nursing care. In 2010, the then Ministry of Health promulgated Hospital Quality Nursing Care Standards (trial) and launched a national Quality Nursing Care Demonstration Project, which set up working standards for public hospitals to enhance nursing care and placed great importance on patient satisfaction with nursing care. In recent years, studies have been carried out to measure the level of patient satisfaction with nursing care and explore its influencing factors such as age, gender, education level, income, health status, and so on [36,37,38,39,40,41,42]. For survey instruments, in addition to single-item measurement, several multi-item instruments were developed [43,44]. However, there were some weaknesses in previous studies. First, some newly-developed instruments were not used in further empirical studies. Second, these studies were mostly conducted in a specific hospital or within a geographic region with a small sample size, and there have been no national-level surveys or studies [45,46,47]. Third, most studies have focused on the influence of patient characteristics on satisfaction and failed to explore the role of hospital and care-related factors [48,49,50].

## 2. Study

### 2.1. Aims

To fill in this research gap, we participated in the survey conducted by the Peking Union Medical College School of Health Policy and Management, and developed and validated a multi-item survey instrument to measure inpatient satisfaction with nursing care and investigated patients in 143 tertiary hospitals across China. We also utilized regression models to explore factors influencing satisfaction, including both patient-related factors and hospital-related factors. This study is an attempt to measure the level of patient satisfaction with hospital nursing care in China and examine key impact factors, to inform policymakers and hospital administrators of fields of improvement, which will contribute to the development of a patient-centered healthcare system.

### 2.2. Design

A correlational, cross-sectional study design was used. The survey instrument for this study was designed based on three multi-item scales: (1) SERVQUAL, which is a widely used scale for measurement of consumer satisfaction, with five domains of tangibles, reliability, responsiveness, assurance, and empathy [51]; (2) patient satisfaction scale (PSS) contains three domains: technical-professional, educational relationship, and trusting relationship; and (3) an indigenous scale designed by Gu for measuring nursing care satisfaction, with five domains: environmental facilities, service quality and outcomes, timely help, health guidance, and humane care [44]. After three rounds of multidisciplinary expert consultation and a small-scale pilot survey, 14 items from five domains were retained (Appendix A). These domains were humane care, professional competence, health guidance, hospital environment, and nurses’ appearance. This patient satisfaction scale was tested using confirmatory factor analysis (CFA) and Cronbach’s alpha, to evaluate the construct validity and internal consistency.

Responses for 14 items were measured on a five-point Likert scale, ranging from 1 to 5, corresponding to “completely unsatisfied”, “unsatisfied”, “neutral”, “satisfied”, and “very satisfied.” In addition to satisfaction domains, the questionnaire included patient characteristics such as age, sex, education, and income. Furthermore, the hospital survey form included general information about the hospitals, such as geographical location, type, and number of doctors and nurses and their professional titles.

### 2.3. Sample

This study used data from the 2021 China National Patient Survey, which involved 24,971 inpatients in 143 hospitals across 31 provinces. The hospital samples consisted of 43 national-level hospitals (including 25 general hospitals and 18 specialist hospitals) affiliated to the NHC (National Health Commission), seven traditional Chinese medicine (TCM) hospitals affiliated to NATCM (National Administration of Traditional Chinese Medicine), and 93 province-level hospitals sampled in 31 provinces (usually 1 general hospital, 1 TCM hospital, and 1 maternal and child health hospital in each province). The patient sample size was at least 1746, to estimate a satisfaction rate of 80% with a precision of plus or minus 2% at the 95% confidence level; and the invalid response rate was set at 10%. Considering the patient volume of the hospitals, a minimum sample size of 150 inpatients was set for each hospital.

### 2.4. Data Collection

Hospital-level data were collected using a self-administered survey form filled in and endorsed by hospital administrative offices. The patient survey was conducted during the period of January to March 2021. Using a convenience sampling method, at least 150 inpatients were recruited in each hospital covering all inpatient departments. Patients on the day of, or the day prior to, discharge, aged 18 and over, and with full consciousness were invited to participate in this survey. They were asked to fill in the questionnaire using their electronic devices under the guidance of trained investigators. All patients provided informed consent for their participation in the study. Finally, a total of 24,971 patients who participated in the survey with effective responses were included in our analysis.

### 2.5. Ethical Considerations

This study was approved by the Ethics Committee(Approval NO CAMS&PUMC-IEC-2021-033). The aim and procedures of the study were fully explained before obtaining oral informed consent from all participants. No names and other personal identifiers were obtained, to ensure confidentiality.

### 2.6. Data Analysis

Based on previous studies and data availability, we selected patient-level factors including age, sex, education, residence, annual household income, and insurance type; and hospital-level factors including hospital affiliation level, geographic location, department, hospital type, nurse-to-doctor ratio, and proportion of senior-titled nurses, as potential impact factors. Hospital nurse-to-doctor ratio reflects the adequacy of nursing personnel, as well as the rationality of hospital staffing, and the proportion of senior-titled nurses is an indicator of the skill level of the nursing staff. Then a *t*-test and variance analysis were performed, to determine the association between patient satisfaction and each potential impact factor. Subsequently, multivariate analysis of the factors associated with inpatient satisfaction was conducted. Due to the heavily skewed distribution of patient satisfaction scores, linear regression methods are not suitable [52]. We then transformed the patient satisfaction score into a dichotomous variable, classifying scores of 4 and above as “satisfied = 1” and scores below 4 as “unsatisfied = 0.” Using the dichotomous variable as the dependent variable, we analyzed the factors associated with patient satisfaction with nursing care through multivariate logistic regression. The data were statistically analyzed using SPSS 23.

### 2.7. Validity and Reliability/Rigour

The patient satisfaction scale was retested with confirmatory factor analysis (CFA) and Cronbach’s alpha, to evaluate the construct validity and internal consistency (Table 1). The KMO was 0.950 (greater than 0.5 required), and the significance of Bartlett’s test was 0.001 (lower than the 0.05 required). Five factors were identified by CFA as explaining 78.374% of the total variance, namely humane care, professional competence, health guidance, hospital environment, and nurses’ appearance, which were consistent with the original domains. Accordingly, all the items in the original domains were retained for the subsequent analysis. We calculated the domain score using the mean of items within each domain. Similarly, the overall inpatient satisfaction score was the average of 14 items.

## 3. Results

### 3.1. Level of Satisfaction

We calculated the average score of items for each domain (Table 2). The overall score was 4.74, and 23,738 patients (95.06%) were satisfied, with a cutoff point of 4. Among the 14 items, the “nice attitude” item received the highest score (4.82), and the “environment quietness” item received the lowest score (4.64). The domain with the highest score was humane care (4.81), and the domain with the lowest score was hospital environment (4.67).

### 3.2. Descriptive and Univariate Analysis for Factors Associated with Inpatient Satisfaction

Descriptive statistics of the sample are presented in Table 3. Among the 24,971 respondents in 143 hospitals, 8562 (34.29%) were in national-level hospitals and 16,409 (65.71%) were in provincial-level hospitals. For hospital type, 10,378 were from general hospitals (41.56%) and 14,593 (58.44%) were from non-general hospitals. For geographic location, the patient distribution in eastern, central, and western areas were 45.57%, 23.86%, and 30.57%, respectively. For department, share of patients in internal medicine, surgery, pediatrics, and others were, respectively, 27.20%, 27.81%, 4.18%, and 40.80%. For nurse-to-doctor ratio and proportion of senior-titled nurses, they were transformed into binary variables using the median as the cutoff point for each variable. For patient demographic characteristics, 14,416 (57.73%) were aged below 50, 15,052 (60.30%) were female, 14,745 (59.05%) had an education level of high school or below, and 16,474 (65.97%) were urban residents. With regard to annual household income, 12,009 (48.09%) had an annual household income below RMB 60,000, 24.48% between RMB 60,000 and 12,000, and 27.43% above RMB 120,000. For insurance, most of the respondents were covered by some kind of social insurance or private insurance, only 2.30% of respondents were uninsured. 

Differences in satisfaction scores between subgroups were analyzed using a *t*-test and variance analysis (Table 3). It was found that patient satisfaction was associated with hospital affiliation level, geographic location, hospital type, department, nurse-to-doctor ratio, age, sex, education, registered permanent residence, annual household income, and insurance. Patients who tended to express higher satisfaction towards nursing care were older than 50 years, male, civil servants, urban residents, enrolled in a government insurance scheme, and had an annual household income over 120,000 RMB; and they sought care in a national level hospital, in eastern areas, in general hospitals, in a department of internal medicine, and in hospitals with higher nurse-to-doctor ratios.

### 3.3. Multivariate Analysis of Factors Associated with Inpatient Satisfaction

A dichotomous variable of patient satisfaction was constructed by considering ≥4 as “satisfied” and <4 as “unsatisfied.” We applied binary logistic regression to explore key factors related to patient satisfaction. The results revealed six determinants of patient satisfaction with nursing care (Table 4).

Regarding patient-level characteristics, the impact factors included age, education, and household income. Compared to older patients aged over 50, younger patients were less satisfied (OR = 1.456). Patients with a college or higher education level were more likely to be satisfied with nursing care than those with a lower education level (OR = 1.208). Compared to patients with an annual household income of less than RMB 60,000, patients with an annual household income between RMB 60,000 and 120,000 (OR = 1.595) and over 120,000 (OR = 1.658) were more likely to be satisfied.

As for hospital-level characteristics, hospital geographic location, type, and nurse-to-doctor ratio significantly impacted patient satisfaction. Patients in hospitals located in the eastern area were more likely to be satisfied with nursing care than patients in hospitals located in the western area (OR = 1.837). When compared to patients in general hospitals, patients in non-general hospitals were less likely to be satisfied with nursing care. Patients in hospitals with higher nurse-to-doctor ratios were more likely to be satisfied than patients in hospitals with lower nurse-to-doctor ratios (OR = 1.177).

## 4. Discussion

Patient satisfaction with nursing care is an important indicator of quality of care and patient prognosis [3,53,54,55]. Our study assessed the level of inpatient satisfaction with nursing care across 143 tertiary hospitals in China and explored factors associated with patient satisfaction at both patient level and hospital level.

In terms of the satisfaction level with nursing care in tertiary hospitals in China, this study found that the overall satisfaction score was 4.74, which is relatively high. This might be because our sample hospitals, which are national or provincial level hospitals, are among the best hospitals in China. These hospitals have better facilities and a higher healthcare quality compared to city- or county-level hospitals [56]. Considering the five domains, “humane care” (4.81) was the domain with the highest satisfaction score, followed by “professional competence” (4.76). “Health guidance” (4.66) was the domain with the lowest satisfaction score. These results are consistent with the results of other studies [57,58]. Nurses play a key role in providing health guidance, which is linked with positive clinical outcomes, such as improved adherence to a therapeutic regime, enhanced ability to cope with symptoms, and healthy lifestyles [9]. However, nurses in China tend to focus on biological factors and patients’ clinical care in hospital and to some extent neglect the importance of health guidance. Furthermore, although some resources and training on health guidance are currently provided to nurses, the majority still lack sufficient knowledge and skills to provide appropriate health guidance. This implies hospitals should provide better health guidance training for nurses.

From the patient side, we found that age, education, and annual household income were significantly associated with inpatient satisfaction. Regarding age, we found that older patients were likely to report higher satisfaction; this may be because younger patients are less compliant with nursing care and more distrustful of paramedics [32,59], whereas older patients are more likely to be compliant and respectful of providers. Regarding education level, patients with a higher level of education tended to be more satisfied with nursing care. Patients with more education may have a higher health literacy. Many studies have shown that patients with limited health literacy are likely to have a more difficulty understanding health information and exchanging information with hospital staff [60,61,62]. Studies also indicated that nurses report more difficulty communicating with low-literacy patients [62]. As a result, educated patients with better health literacy feel more satisfied with the care provided. Lastly, we found that, in agreement with previous studies, patients with higher incomes reported higher satisfaction [63]. This might be because patients with higher incomes may choose better wards, or employ more self-paid nurse aids to assist with care, so they tend to be more satisfied. Thus, it is necessary to pay more attention and give more support to low-income patients. The difference of satisfaction among different patient groups implies that the evaluation of patient satisfaction across hospitals should consider the composition of patients [8,64,65,66].

From the hospital side, we have found that geographic location, type, and nurse-to-doctor ratio were significantly associated with inpatient satisfaction with nursing care. In terms of geographic location, patients in relatively affluent regions (eastern and central regions) tended to be more satisfied than patients in the western region. China is a country with great disparities among regions. In 2020, the per capita GDP of Beijing in the eastern region was 3.75 times as high as that of Guangxi in the western region [67]. In economically advanced areas, hospitals can obtain more funding from local governments and are likely to have more advanced facilities and equipment and better-trained health professionals, leading to higher patient satisfaction [68]. Policies are needed to reduce regional disparities in healthcare resource allocation, to improve patient outcomes.

Considering the type of hospital, compared to inpatients in general hospitals, those in non-general hospitals were less satisfied with nursing care. Previous studies found that patient satisfaction varied across different hospital types [35,69,70]. General hospitals in China are usually medical centers within administrative territories and have better government support, facilities, and management. Previous studies have identified that the comprehensive performance (including nursing care) of general hospitals is higher than that of non-general hospitals [71]. This implies that priority should be given to the improvement of nursing care and overall performance of non-general hospitals, including TCMs, MCHs, and other specialized hospitals.

Furthermore, the results indicate that a higher nurse-to-doctor ratio is beneficial for increasing patient satisfaction with nursing care, which is consistent with the findings in previous studies [72,73,74,75,76]. Although a standard set by the government in 1989 stated that hospitals should have a nurse-to-doctor ratio of no less than 2:1 [77], over 95% of hospitals in this survey had a nurse-to-doctor ratio of less than 2:1, which is significantly lower than that of developed countries, such as Canada (5.3:1), England (3.8:1), and the USA (3.7:1) [78]. Evidence suggests that inadequate nurse staffing leads to heavy workload and widespread job burn-out among nurses, which may lead to an increase in adverse events, and so is documented as a serious threat to the quality of care [64,79,80]. The government should work on growing the nurse workforce and significantly increasing the nurse-to-doctor ratio in hospitals. Besides recruitment, it is also important to improve the retention of nurses by adopting effective policies, such as offering better compensation packages and providing broader career paths. In addition, the duties of nurses and physicians need to be clearly defined and distinguished, and qualified nurses need to be given more responsibilities in patient care.

## 5. Limitations

The limitations of this study need to be acknowledged to facilitate future improvement. First, the hospitals in this study included only tertiary public hospitals and excluded secondary and primary hospitals, as well as private hospitals. Therefore, the results cannot represent the general level of patient satisfaction with nursing care in Chinese hospitals. Second, inpatient respondents were surveyed before they were discharged from hospitals, which may have led to bias in their responses (more positive responses to satisfaction questions). Lastly, although this study explored several hospital-level factors as impact factors (i.e., geographic location, type, department, nurse-to-doctor ratio, and ratio of senior job titles), there are other important factors that were not included due to lack of data; for example, hospital work environment, information exchanges, and specialty training for care providers. These factors should be addressed in future research.

## 6. Conclusions

This study assessed patient satisfaction with nursing care in Chinese tertiary hospitals using a self-developed multi-item questionnaire. It is the first study for patient satisfaction with nursing care based on a nationally representative survey and sample. Factors influencing satisfaction, including both patient-related factors and hospital-related factors were explored, which offered some unique insights for the improvement of nursing care and patient satisfaction in tertiary hospitals across China. The results suggest that policymakers need to pay close attention to equitably allocating healthcare resources among different regions, improving the management and competence of non-general hospitals, and strengthening the recruitment and retention of the nursing workforce. Furthermore, patient characteristics should be considered in the evaluation of patient satisfaction across hospitals. These findings can be used to develop targeted interventions aimed at the development of a patient-centered healthcare system.

## Figures and Tables

**Table 1:** Rotated components matrix from CFA (factor loadings).

Items	Factor (Cronbach’s Alpha)				
	Humane Care (0.862)	Professional Competence (0.927)	Health Guidance (0.802)	Hospital Environment (0.738)	Nurses’ Appearance (−)
Respect for patients	0.398	−0.128	−0.11	−0.03	−0.023
Concern for illness	0.389	−0.139	−0.081	−0.031	−0.03
Privacy protection	0.384	−0.127	−0.083	−0.035	−0.033
Nice attitude	0.373	−0.133	−0.073	−0.031	−0.019
Sense of responsibility	−0.24	0.535	−0.039	−0.071	−0.061
Nursing skills	−0.105	0.426	−0.088	−0.048	−0.022
Timely help	−0.058	0.399	−0.125	−0.036	−0.025
Attention to discomfort	−0.033	0.363	−0.124	−0.025	−0.005
Medication instructions	−0.109	−0.124	0.615	−0.145	−0.016
Informing about precautions	−0.144	−0.124	0.563	−0.01	−0.032
Disease knowledge education	−0.097	−0.047	0.482	−0.092	−0.015
Environment quietness	−0.071	−0.094	−0.135	0.714	−0.041
Environment comfortableness	−0.052	−0.064	−0.09	0.606	−0.046
Pleasing appearance	−0.064	−0.071	−0.04	−0.056	1.062

Note: The overall internal consistency of 14 items was represented by a Cronbach’s alpha = 0.93.

**Table 2:** Satisfaction scores of patients with nursing care for each domain and item.

Domains	Items	Satisfaction Scores	
Humane care			4.81
	Respect for patients	4.81	
	Concern for illness	4.80	
	Privacy protection	4.80	
	Nice attitude	4.82	
Professional competence			4.76
	Sense of responsibility	4.69	
	Nursing skills	4.77	
	Timely help	4.78	
	Attention to discomfort	4.80	
Health guidance			4.66
	Medication instructions	4.66	
	Informing about precautions	4.66	
	Disease knowledge education	4.69	
Hospital environment			4.67
	Environment quietness	4.64	
	Environment comfortableness	4.71	
Nurses’ appearance	Pleasing appearance	4.72	4.72
Total score	4.74		

**Table 3:** Characteristics of respondents and overall satisfaction towards nursing care of subgroups (*n* = 24,971).

Factors	Number of Patients (%)	Satisfaction Scores (M ± SD)	Test Statistic	*p*
Hospital Affiliation level				
National-level	8562 (34.29%)	4.55 ± 0.34	t = 6.472	**<0.001** ***
Provincial-level	16,409(65.71%)	4.51 ± 0.36		
Geographic location				
Eastern area	11,379 (45.57%)	4.78 ± 0.35	F = 189.701	**<0.001** ***
Central area	5959 (23.86%)	4.75 ± 0.35		
Western area	7633 (30.57%)	4.67 ± 0.42		
Hospital Type				
General	10,378 (41.56%)	4.77 ± 0.35	t = 10.314	**<0.001** ***
Non-general (TCM/MCH/others)	14,593 (58.44%)	4.75 ± 0.39		
Department				
Internal medicine	6793 (27.20%)	4.78 ± 0.38	F = 3.449	**0.016** *
Surgery	6944 (27.81%)	4.74 ± 0.37		
Pediatrics	1045 (4.18%)	4.75 ± 0.37		
Others	10,189 (40.80%)	4.74 ± 0.37		
Hospital nurse-to-doctor ratio				
Low (≤1.497)	12,541 (50.22%)	4.73 ± 0.38	t = −4.771	**<0.001** ***
High (>1.497)	12,430 (49.78%)	4.75 ± 0.37		
Ratio of senior-titled nurses in the hospital				
Low (≤3.413)	12,539 (50.21%)	4.73 ± 0.37	t = −2.296	0.111
High (>3.413)	12,432 (49.79%)	4.74 ± 0.37		
Age				
≤50	14,416 (57.73%)	4.73 ± 0.38	t = −3.975	**<0.001** ***
>50	10,555 (42.27%)	4.75 ± 0.36		
Sex				
Male	9913 (39.70%)	4.74 ± 0.37	t = 1.907	**0.006** **
Female	15,058 (60.30%)	4.74 ± 0.38		
Education				
College or above	10,226 (40.95%)	4.75 ± 0.36	t = 5.494	**<0.001** ***
High school or below	14,745 (59.05%)	4.73 ± 0.38		
Registered permanent residence				
Rural	8497 (34.03%)	4.72 ± 0.39	t = −6.310	**<0.001** ***
Urban	16,474 (65.97%)	4.75 ± 0.36		
Annual household income (RMB)				
0–60,000	12,009 (48.09%)	4.71 ± 0.40	F = 94.082	**<0.001** ***
60,000–120,000	6112 (24.48%)	4.75 ± 0.35		
>120,000	6850 (27.43%)	4.78 ± 0.33		
Insurance				
GIS	1525 (6.11%)	4.77 ± 0.35	F = 5.774	**<0.001** ***
UEBMI	10,267 (41.12%)	4.74 ± 0.36		
RBMI	11,731 (46.98%)	4.73 ± 0.38		
Others	874 (3.50%)	4.72 ± 0.41		
No insurance	574 (2.30%)	4.72 ± 0.41		

Abbreviations: *t*, test statistic of independent sample *t*-test; F, test statistic of variance analysis; GIS, government insurance scheme; UEBMI, urban employees basic medical insurance; RBMI, urban and rural residents basic medical insurance; TCM, traditional Chinese medicine; MCH, maternal and child health hospital. Bold values indicate statistical significance as determined by *p* < 0.05. * *p* < 0.05, ** *p* < 0.01, *** *p* < 0.001.

**Table 4:** Binary logistic regression of overall satisfaction with patient and hospital characteristics (OR with 95% confidence interval).

Variables	B	SE	Wald χ²	*p*	OR	95% CI
Geographic location						
Western area (reference)						
Eastern area	0.608	0.08	58.04	**<0.001** ***	1.837	(1.571, 2.148)
Central area	0.597	0.072	68.548	**<0.001** ***	1.817	(1.577, 2.093)
Type						
General (reference)						
Non-general (TCM/MCH/others)	−0.276	0.07	15.383	**<0.001** ***	0.759	(0.661, 0.871)
Nurse-to-doctor ratio						
Low (reference)						
High	0.163	0.066	6.18	**0.013** **	1.177	(1.035, 1.339)
Age						
≤50 (reference)						
>50	0.376	0.069	29.983	**<0.001** ***	1.456	(1.273, 1.666)
Education						
High school or below (reference)						
College or above	0.189	0.069	7.433	**0.006** **	1.208	(1.055, 1.384)
Annual household income (RMB)						
0–60,000 (reference)						
60,000–120,000	0.467	0.079	35.039	**<0.001** ***	1.595	(1.366, 1.861)
>120,000	0.506	0.081	38.547	**<0.001** ***	1.658	(1.413, 1.945)
Constant	1.804	0.114	249.056	**<0.001** ***	6.076	

Abbreviations: TCM, traditional Chinese medicine; MCH, maternal and child health hospital. Bold values indicate statistical significance as determined by *p* < 0.05. ** *p* < 0.01, *** *p* < 0.001.

## Data Availability

The data that support the findings of this study are available from the corresponding author upon reasonable request. The data are not publicly available due to privacy restrictions.

## Appendix A. Patient Satisfaction Scale with Nursing Care

**Items**	**Completely Unsatisfied**	**Unsatisfied**	**Neutral**	**Satisfied**	**Very Satisfied**
The nurses treat me with respect.					
The nurses care about my conditions and needs.					
The nurses protect my privacy.					
The nurses have friendly and kind attitude.					
The nurses are responsible for their work.					
The nurses are skillful.					
The nurses are available when I need support.					
The nurses check on and keep track of my conditions when I feel uncomfortable.					
The nurses show me how to take medication.					
The nurses inform me enough about precautions.					
The nurses give me education about my disease.					
The environment of the wards is quiet.					
The environment of the wards is comfortable.					
The nurses dress appropriately and neatly.					
